# Editorials

**Published:** 1893-09

**Authors:** 


### HOMŒOPATHIC PHYSICIAN,

A MONTHLY JOURNAL OF

HOMŒOPATHIC MATERIA MEDICA AND CLINICAL MEDICINE.

"If our school ever gives up the strict inductive method of Hahnemann, we are lost, and deserve only to be mentioned as a caricature in the history of medicine."—CONSTANTINE HERING.

Vol. XIII. SEPTEMBER, 1893. No. 9.

### EDITORIALS.

NEURAL ANALYSIS.—There seems to be some want of understanding among the readers of this journal as to the nature of the instrument by which Prof. Jaeger has made his elaborate experiments in neural analysis. The apparatus consists of a stop-clock or chronoscope of peculiar construction, capable of registering thousandths of a second. The clock being started the problem is to stop the motion of the hands in the shortest possible time. This is done by pressing an electric button. The time required to do this will vary with the physical and mental condition of the individual who performs the experiment.

A full description of the instrument may be found in *The Hahnemannian Monthly* for May, 1881. It may also be found in *The Medical Advance* for March, April, and May of the present year.

One of the instruments may be seen in the office of the editor of this journal.

Lately Prof. Jaeger has been using a pocket chronoscope which looks like a watch with the exception of the face, which is divided into a great number of small divisions, and has only a single hand. If a photograph of the instrument can be procured it will be published in these pages, together with an explanation.

Dr. Wells on Intermittent Fever.—In this number of The Homœopathic Physician the publication of the second part of Dr. Wells' book on *Intermittent Fever* is resumed. The first part is already in the hands of the subscribers. The continued publication of the work was abandoned because being a translation of Bœnninghausen's well-known work it was considered to be an infringement of the excellent translation of Dr. Korndœrfer. A number of inquiries having been made of the editor for this second part, and the consent of Messrs. Bœricke & Tafel, the publishers of Dr. Korndœrfer's translation, having been kindly given, it has been decided to issue the second part, and so bring the book to a finish.

